# Comparative Proteomics Reveals the Anaerobic Lifestyle of Meat-Spoiling *Pseudomonas* Species

**DOI:** 10.3389/fmicb.2021.664061

**Published:** 2021-04-06

**Authors:** Sandra Kolbeck, Miriam Abele, Maik Hilgarth, Rudi F. Vogel

**Affiliations:** ^1^Lehrstuhl für Technische Mikrobiologie, Technische Universität München, Freising, Germany; ^2^Bayerisches Zentrum für Biomolekulare Massenspektrometrie (BayBioMS), Freising, Germany

**Keywords:** arginine fermentation, anaerobic lifestyle, proteomics, redox stress, modified atmosphere packaging, meat spoilage, *Pseudomonas*, Entner-Doudoroff pathway

## Abstract

The ability of certain *Pseudomonas* (*P.*) species to grow or persist in anoxic habitats by either denitrification, acetate fermentation, or arginine fermentation has been described in several studies as a special property. Previously, we had isolated strains belonging to the species *P. lundensis*, *P. weihenstephanensis*, and *P. fragi* from anoxic modified atmosphere packaged (MAP) minced beef and further proved their anaerobic growth *in vitro* on agar plates. This follow-up study investigated the anaerobic growth of two strains per respective species *in situ* on inoculated chicken breast filet under 100% N_2_ modified atmosphere. We were able to prove anaerobic growth of all six strains on chicken breast filet with cell division rates of 0.2–0.8/day. Furthermore, we characterized the anaerobic metabolic lifestyle of these *Pseudomonas* strains by comparative proteomics, upon their cultivation in meat simulation media, which were constantly gassed with either air or 100% N_2_ atmospheres. From these proteomic predictions, and respective complementation by physiological experiments, we conclude that the *Pseudomonas* strains *P. fragi*, *P. weihenstephanensis*, *P. lundensis* exhibit a similar anaerobic lifestyle and employ arginine fermentation via the arginine deiminase (ADI) pathway to grow anaerobically also on MAP meats. Furthermore, glucose fermentation to ethanol via the ED-pathway is predicted to enable long term survival but no true growth, while respiratory growth with nitrate as alternative electron acceptor or glucose fermentation to acetate could be excluded due to absence of essential genes. The citric acid cycle is partially bypassed by the glyoxylate shunt, functioning as the gluconeogenetic route without production of NADH_2_ under carbon limiting conditions as e.g., in packaged meats. Triggered by an altered redox balance, we also detected upregulation of enzymes involved in protein folding as well as disulfide bonds isomerization under anoxic conditions as a counteracting mechanism to reduce protein misfolding. Hence, this study reveals the mechanisms enabling anaerobic grow and persistence of common meat-spoiling *Pseudomonas* species, and further complements the hitherto limited knowledge of the anaerobic lifestyle of *Pseudomonas* species in general.

## Introduction

The genus *Pseudomonas* currently comprises more than 240 valid published and correct named species (Parte, 2018; Parte et al., 2020), out of which several species have been identified as key players of food spoilage: *P. fluorescens*, *P. putida*, *P. fragi*, *P. lundensis*, and *P. weihenstephanensis* are spoiling meat (Ercolini et al., 2007; Hilgarth et al., 2019) but also cheese and raw milk (Wiedmann et al., 2000; Morales et al., 2005; Marchand et al., 2009; Martin et al., 2011; Scatamburlo et al., 2015; Neubeck et al., 2016; Meng et al., 2017). Their high spoilage potential is due to proteolytic and lipolytic enzymes, which are released out of the cell, initiating changes in the physicochemical and organoleptic properties of products (Alquati et al., 2002; Rajmohan et al., 2002; Marchand et al., 2009; Stoeckel et al., 2016).

Most *Pseudomonas* species are described as obligately aerobic microorganisms, which are unable to grow under conditions of complete oxygen exclusion (Garrity et al., 2005). Thus, anoxic food packaging technologies such as vacuum and modified atmosphere packaging (MAP) are powerful tools to limit spoilage by *Pseudomonas* species (Wang et al., 2018). Nevertheless, it is known that some *Pseudomonas* species such as *P. aeruginosa* and *P. denitrificans* are able to grow even under strictly anoxic conditions (O_2_ < 1 ppm) by either usage of alternative electron acceptors (mainly nitrate respiration) (Carlson and Ingraham, 1983), arginine fermentation (Vander Wauven et al., 1984) or pyruvate fermentation (Eschbach et al., 2004). Reports in the literature of anaerobic growth of meat-spoiling *Pseudomonas* spp. are scarce. A study by Wang et al. (2017) demonstrated anaerobic growth of several *P. fragi* strains in tryptone soy broth media. Other studies identified *P. fragi* strains in vacuum packed (VP) beef samples (Ercolini et al., 2011; Jääskeläinen et al., 2016), whereas De Filippis et al. (2019) proved those strains to be viable and metabolically active on VP beef, although those characteristics were strongly strain dependent (De Filippis et al., 2019).

Furthermore, we have previously investigated the diversity development of *Pseudomonas* spp. during minced beef spoilage and isolated certain anaerobic *P. fragi*, *P. lundensis* and *P. weihenstephanensis* strains (Hilgarth et al., 2019). Although these species had been previously described as strictly aerobic at that time, that study could demonstrate anaerobic growth for isolates of the three species *in vitro* on agar plates. However, the potential of those meat-spoiling *Pseudomonas* species to either grow or only persist under anoxic conditions has not yet been investigated in detail and the underlying mechanism remained unknown in the previous work. Consequently, this study addresses this question by performing a full-proteomic study in meat simulation media, providing insights into the aerobic and anaerobic metabolism of the isolated *Pseudomonas* strains from Hilgarth et al. (2019). Furthermore, we checked the *in situ* growth of all strains on anoxically packaged chicken meat (100% N_2_).

## Materials and Methods

### Strain Selection

Two strains of each meat-spoiling species *P. lundensis* (TMW2. 1732/TMW2.2076), *P. weihenstephanensis* (TMW2.2077/TMW2. 1728), and *P. fragi* (TMW2.2081/TMW2.2082) were chosen for this study. All strains were isolated in previous studies from minced meat (Hilgarth et al., 2019) or beef steak (Hilgarth et al., 2018a) initially packaged under modified atmosphere (>70% O_2_, >20% CO_2_). Isolation of anaerobically growing *Pseudomonas* strains has been performed at late storage time of the meat products when oxygen had already been depleted within the packages. *P. aeruginosa* DSM 1117 was used as a positive control for anaerobic nitrate respiration and substrate utilization tests.

### DNA Extraction and Sequencing

DNA extraction was performed using the E.Z.N.A. bacterial DNA Kit (Omega Bio-Tek, Norcross, GA) following the manufacturer’s instructions. Isolated DNA was sent for sequencing to the microbiome core facility of the Institute of Food & Health (TUM Freising, Germany) for Illumina MiSeq shotgun sequencing.

The NCBI accession numbers for all strains are as followed: *P. lundensis* TMW2.1732 (JACJCQ000000000), *P. lundensis* TMW2.2076 (JAAEBS000000000), *P. weihenstephanensis* TMW2.2077 (JAAEBW000000000), *P. weihenstephanensis* TMW2.1728 (JAAEBV000000000), *P. fragi* TMW2.2081 (JAAEBQ 000000000), and *P. fragi* TMW2.2082 (JAAEBR000000000).

### Test of Anaerobic Growth *in situ* on Chicken Breast

Chicken breast filet, packaged under a modified atmosphere containing 70% O_2_ and 30% CO_2_ were obtained from a local discounter with a use-by date assigned to >3 days. Packages were opened and meat was cut into equally thick, square pieces (36 cm^2^) under sterile conditions. For meat inoculation, cells were pre-cultured oxically, overnight in brain heart infusion (BHI) media (Roth, Karlsruhe, Germany) at 25°C. Before harvesting, cultures were adapted to chilled conditions by shaking at 4°C for 4 h. Afterward, cells were washed twice with quarter-strength ringer’s solution (Merck, Darmstadt, Germany). The prepared meat pieces were inoculated on both sides with 100 μl of OD_600_ = 10 of one of the strains. Cells were distributed homogeneously on the meat surface using a sterile spatula. Inoculation was performed at high cell counts (approx. log_10_/cm^2^ = 6) to outcompete the autochthonous microbiome of purchased chicken meat, which was log_10_/cm^2^ = 2.0–4.9, depending on the package. Inoculated meat pieces were packaged under an oxygen free atmosphere (100% N_2_) within polypropylene (PP) trays (ES-Plastic, Hutthurm, Germany) coated with an oxygen impermeable barrier film ethylen-vinylalcohol (EVOH) (oxygen transmission rate 0.5 cc/m^2^/24 h) using the packaging machine Rotarius VG (VarioVAC, Zarrentin, Germany). CO_2_ in the MAP (as mostly used in industrial packages) was omitted to avoid any additional effects of this gas and strictly focus on the anoxic conditions effect. To ensure complete anoxic conditions, an oxygen scavenger AnaeroGen 2.5 liter (Thermo Fisher Scientific, Waltham, MA) was added to each package, containing three meat pieces (3 replicates). Packages were stored at 4°C until sampling.

Samples for CFU determination were taken after 0, 3, 5, and 7 days. Packages were opened within a Bactron anaerobic chamber (Sheldon, Cornelius, United States) containing <5% H_2_ and >95% N_2_ atmosphere. Each meat piece was transferred into a sterile 50 ml falcon tube containing quarter-strength ringer’s solution and homogenized by vertexing for 2 min. Afterward, the cell suspension was diluted serial times and each dilution was plated on BHI plates. Cultivation of plates was performed anoxically at 25°C for 48 h. Significant growth on meat was checked by a *t*-test between log_10_ (CFU/cm^2^) values obtained at day 0 with CFU values of the other days (day 0–3, day 0–5, and day 0–7; significance level *p* < 0.05). The recovery/identity of inoculated strains on meat was proven by a random amplified polymorphic DNA (RAPD) PCR (Hilgarth et al., 2018b), performed with 12 colonies of each replicate after 7 days.

### Experimental Design of the Proteomic Study

In order to exclude interfering background microbiota and high numbers of detected meat proteins, all strains were cultivated *in vitro* in sterile glass bottles containing 400 ml of a meat simulation (MS) media as previously employed and described by Kolbeck et al. (2019). In detail, MS media consists of 12.5 g/l meat extract, 0.05 mM Tween80, 0.5% glycerol and 2 μg/ml hemin chloride adjusted to a pH of 5.8 with 100% lactic acid. MS medium was inoculated with an optical density of 0.1 at 590 nm with freshly prepared and washed precultures as described above. Bacteria were cultivated for 48 h, 120 rpm at 25°C ± 2°C. During cultivation, MS media were constantly aerated with either 100% N_2_ or air. Growth of the strains was monitored over whole cultivation period and samples for proteomic were taken at logarithmic growth phase.

### Proteomic Sample Preparation, Measurement, and Data Analysis

Samples for the proteomic study were prepared as described previously (Kolbeck et al., 2020). Briefly, cells were lysed, protein concentrations were determined; 100 μg proteins were reduced, carbamidomethylated and digested with trypsin. The resulting peptide mixture was desalted and finally resuspended to a concentration of 0.1 μg^∗^μl^–1^. LC-MS/MS data acquisition was performed on an Ultimate 3000 RSLCnano system coupled to a Q-Exactive HF-X mass spectrometer (Thermo Fisher Scientific, Waltham, MA). All operating parameters were set equal to Kolbeck et al. (2020). Identification and quantification of proteins was done by the software MaxQuant (version 1.6.3.4) (Tyanova et al., 2016a) using its built-in search engine Andromeda (Cox et al., 2011). All build in options and operating parameters of MaxQuant were set equal as described by Kolbeck et al. (2020).

### Proteomic Data Interpretation

The open-source software Perseus (Tyanova et al., 2016b) was used for statistical data analysis. Therefore, proteins were filtered for high-confidence (false-discovery rate cutoff < 1% based on peptide-spectrum match (PSM) level and protein level, removal of reverse, contaminant and “only-identified by site” proteins) and grouped into biological replicates. Label free quantification (LFQ) values were log_2_ transformed and a Welch *t*-test was performed revealing statistic significantly differential regulated proteins between the two cultivation conditions air and N_2_ (*p* < 0.01 and log_2_ > 2). All obtained proteins were further confirmed by manual sequence searches using the basic local alignment search tool (BLAST) provided by NCBI (Altschul et al., 1990). Different annotation programs (NCBI, TIGR, SEED, KEGG) as well as manual curation was applied to group the identified proteins into functional categories, revealing whole metabolic pathways being differentially regulated between oxic and anoxic cultivation of our *Pseudomonas* strains.

### API 20 NE Test

An API NE 20 test (BioMérieux, Nürtingen, Germany) was performed following the manufacturer’s instructions. Results from the NO_3_, TRP, Glu were read out after 24 h, while other results were read out after 48 h of incubation of the stripe.

### *In vitro* Test for Anaerobic Arginine and Glucose Fermentation

Arginine and glucose metabolism were checked performing a plate assay. Therefore, 20 mM L-arginine (Sigma-Aldrich GmbH, Darmstadt, Germany) or 20 mM glucose monohydrate (Merck, Darmstadt, Germany) was supplemented to 5 g^∗^l^–1^ NaCl (Roth, Karlsruhe, Germany), 2 g^∗^l^–1^ KH_2_PO_4_ (Merck, Darmstadt, Germany), 1 g^∗^l^–1^ peptone from soy (Roth, Karlsruhe, Germany) and 12 mg^∗^l^–1^ phenol red (Thermo Fisher Scientific, Waltham, MA). The latter was added as a pH indicator, changing from yellow to red-purple in case of alkalization, as expected from arginine degradation. All chemicals were added after autoclaving by sterile filtering with 0.02-mm pore size. The final pH was adjusted to 6.0. Medium without arginine or glucose monohydrate was used as control.

*Pseudomonas* strains were precultured in BHI as described above, washed twice in sterile quarter-strength ringer’s solution and adjusted to an OD_600_ = 0.5. 20 μl of the prepared precultures were dripped on plates containing arginine and no arginine or glucose and no glucose. The experiment was performed twice, as plates were cultivated once under oxic and once under anoxic conditions at 25°C. Anoxic plates were packaged into sterile PP-EVOH trays with 100% N_2_ containing an oxygen scavenger as described above. After 4 days, color change was validated taking standardized pictures using a colonyDoc-It imaging station (VWR, Darmstadt, Germany). *P. aeruginosa* DSM 1117 was used as a positive control.

### *In vitro* Test for Anaerobic Nitrate Respiration

Dissimilatory nitrate reduction was checked by a physiological assay, monitoring growth of our *Pseudomonas* strains in presence or absence of NO_3_^2–^. Therefore, a minimal medium was prepared containing 4.75 g^∗^l^–1^ K_2_HPO_4_ (Merck, Darmstadt, Germany), 4.55 g^∗^l^–1^ KH_2_PO_4_, 2 g^∗^l^–1^ yeast extract (Roth, Karlsruhe, Germany) and 20 mM glucose monohydrate. Medium was eighter supplemented with 20 mM NaNO_3_ or no NaNO_3_ and pH was adjusted to 6.69. After autoclaving, media was aliquoted into microplates and kept in an anaerobic chamber for 48 h to become anoxic prior to inoculation. Plates were inoculated with an OD_600_ = 0.1 of a freshly prepared preculture as described above. Growth was measured using a Fluostar Omega microplate reader (BMG LABTECH GmbH, Ortenberg, Germany) after 3 days of incubation within the anaerobic chamber at 25°C. *P. aeruginosa* DSM 1117 was used as a positive control for nitrate respiration. Significant growth differences were defined based on a two-side open *t*-test (*p* < 0.01) between samples containing NaNO_3_ and no NaNO_3_.

## Results

### *In situ* Growth on Anoxically Packaged Chicken Breast Filet

**FIGURE 1:**
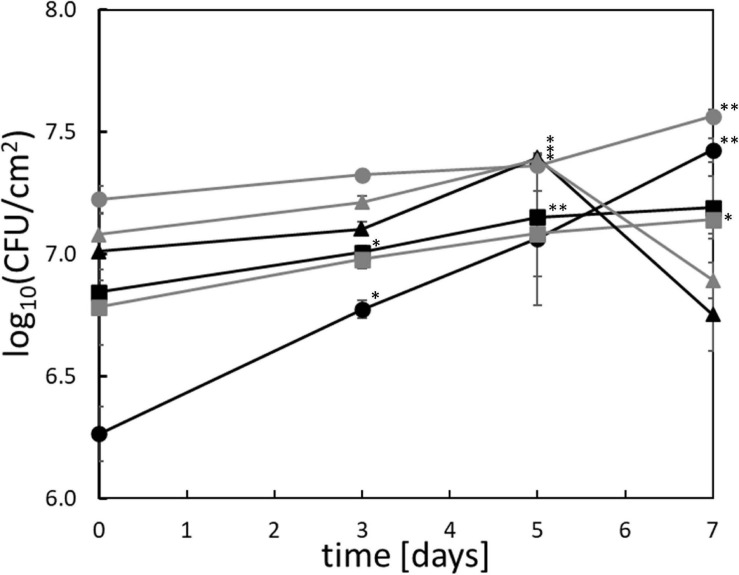
Anaerobic growth of *Pseudomonas in situ* on chicken meat. The six strains (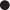) *P. lundensis* TMW2.1732, (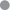) *P. lundensis* TMW2.2076, (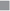) *P. weihenstephanensis* TMW2.2077, (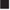) *P. weihenstephanensis* TMW2.1728, (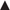) *P. fragi* TMW2.2081, and (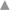) *P. fragi* TMW2.2082 were checked for their anaerobic growth on chicken breast filet over 7 days at 4°C. ^∗^significant (p = 0.05–0.01) or ^∗∗^highly significant (p ≤ 0.01) log_10_(CFU/cm^2^) compared to the log_10_(CFU/cm^2^) on day 0. SE = standard error based one three independent replicates.

All tested *Pseudomonas* strains exhibited significant but limited anaerobic growth on chicken breast after >3 days of cultivation at 4°C (Figure 1). *P. lundensis* TMW2.1732 and *P. weihenstephanensis* TMW2.1728 exhibited significant growth after 3 days, *P. lundensis* TMW2.2076 and both *P. fragi* strains after 5 days and *P. weihenstephanensis* TMW2.2077 after 7 days. The cell division rates were low for all *Pseudomonas* strains (0.4/d) except for *P. lundensis* TMW2.1732, which exhibited a cell division rate of 0.8/d.

### Growth and Comparative Proteomic Analysis of *Pseudomonas* spp. *in vitro* in MS Media Under Oxic and Anoxic Conditions

Growth in MS-medium was determined by monitoring the optical density (OD = 600 nm) (Supplementary Figure 1). Similar to growth on meat, the anoxically growth in MS media was also limited for all strains, reaching a maximal OD_600_ of 0.22. Most strains, except for *P. fragi* TMW2.2081, began to enter stationary phase after 48 h of anoxic cultivation. Furthermore, an increase in pH values of the MS-media of approx. 0.65 was detected within 48 h under anoxic cultivation for all strains analyzed (Supplementary Figure 2).

During exponential growth phase, samples for proteomic analysis were taken. Approximately 53–57% of the genomically predicted proteins could be detected by the mass spectrometer for each of the strains (Table 1). Both *P. weihenstephanensis* strains and *P. fragi* TMW2.2082 exhibited more than twice as much significantly regulated proteins compared to *P. fragi* TMW2.2081 and both *P. lundensis* strains. *P. fragi* TMW2.2081 was the strain with the lowest number of regulated proteins under the conditions tested. Overall, a high degree of regulation under the different atmospheres was detected for all *Pseudomonas* strains. A detailed list of differentially expressed single enzymes for each strain is provided in Supplementary Table 1.

**TABLE 1:** Summary of the proteins identified by our proteomic study.

	TMW	Encoded	Detected	Under anoxic conditions significantly		Total
				upregulated	downregulated	
*P. lundensis*	2.1732	4.523	2,450	47	50	**97**
*P. lundensis*	2.2076	4.215	2,380	30	56	**86**
*P. weihenstephanensis*	2.2077	4.599	2,585	168	109	**277**
*P. weihenstephanensis*	2.1728	4.270	2,441	156	68	**224**
*P. fragi*	2.2081	4,565	2,440	25	50	**75**
*P. fragi*	2.2082	4,571	2,451	153	103	**256**

*List of total amounts of genomic encoded proteins (based on NCBI), MS detected proteins (after MaxQuant search) and significantly (p < 0.01, log_2_ > 2) up- or downregulated proteins under anoxic conditions (after Perseus analysis) of the six strains P. lundensis TMW2.1732, P. lundensis TMW2.2076, P. weihenstephanensis TMW2.2077, P. weihenstephanensis TMW2.1728, P. fragi TMW2.2081, and P. fragi TMW2.2082.*

Strains of all three *Pseudomonas* species exhibited similar differential proteomic expression patterns within the relevant major metabolic pathways indicating genus specific metabolic reaction. A detailed list of those metabolic pathways and their direction of regulation is provided in Table 2 for all strains. Important metabolic pathways regarding their energy metabolism were genomically predicted (Supplementary Table 2) and illustrated in separate figures, which further highlight the genus specific metabolic regulations according to proteomic data.

**TABLE 2:** Summary of the main differentially regulated metabolic pathways.

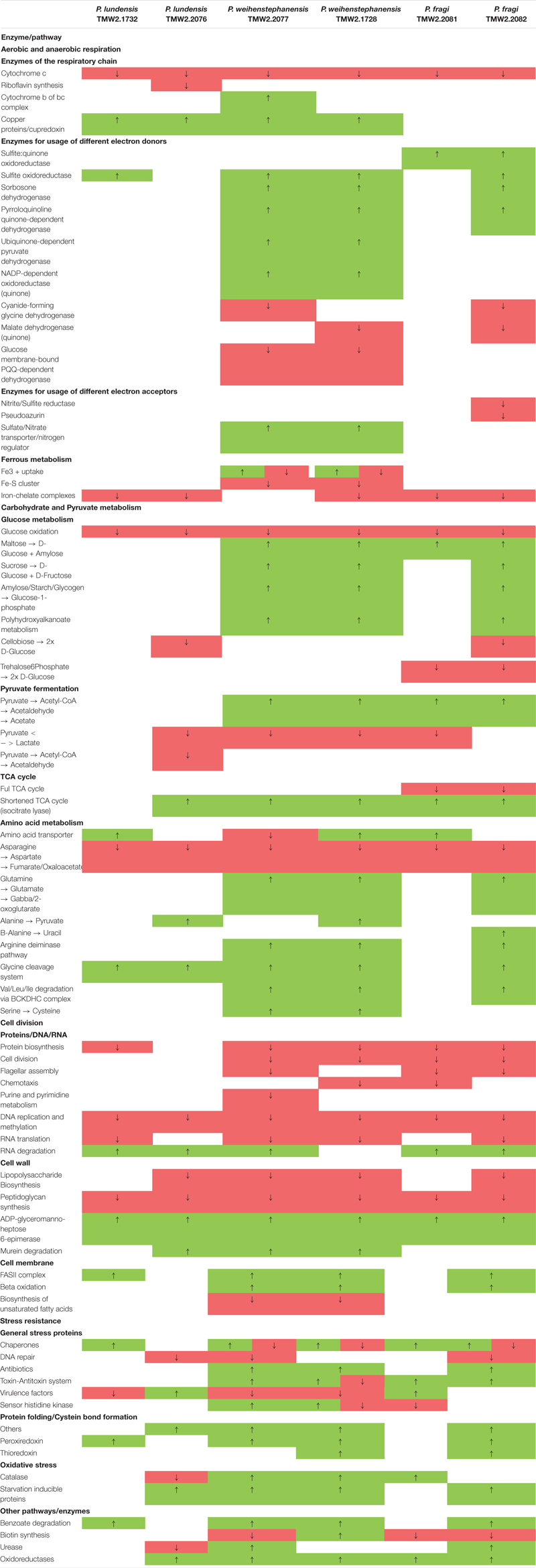

*This list summarizes main differential regulated metabolic pathways of the strains P. lundensis TMW2.1732, P. lundensis TMW2.2076, P. weihenstephanensis TMW2.2077, P. weihenstephanensis TMW2.1728, P. fragi TMW2.2081, and P. fragi TMW2.2082 grown under air and 100% N_2_ atmosphere. Green arrows: the metabolic pathway was upregulated under anoxic conditions, Red arrow: the metabolic pathway was downregulated under anoxic conditions. All arrows are based on single enzyme regulations which can be seen in the Supplementary Table 1.*

**FIGURE 2:**
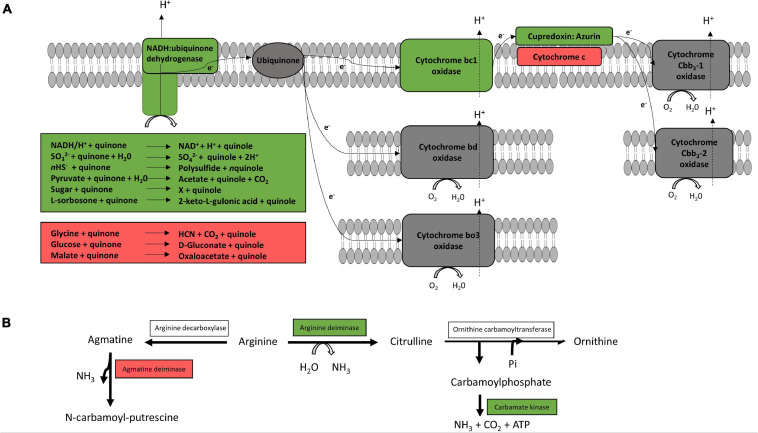
Predicted respiratory chain and arginine metabolism. The respiratory chain setup **(A)** and arginine metabolism **(B)** of the six strains *P. lundensis* TMW2.1732, *P. lundensis* TMW2.2076, *P. weihenstephanensis* TMW2.2077, *P. weihenstephanensis* TMW2.1728, *P. fragi* TMW2.2081, and *P. fragi* TMW2.2082 were predicted based on our genomic study and differentially expressed enzymes were marked. Enzymes marked in green were upregulated and enzymes marked in red downregulated under anoxic conditions for at least one strain.

Figure 2A shows the genomically predicted respiratory chain of our *Pseudomonas* strains, which was similar to the previously described respiratory chain of *P. aeruginosa* by Liang et al. (2020). All cytochromes described for *P. aeruginosa*, except cytochrome caa_3_, could be identified in the genomes of our species. However, neither terminal oxidases cytochrome bd (CIO) and cytochrome bo_3_ (Cyo) with a low oxygen affinity nor terminal oxidases Cbb_3_-1 and Cbb_3_-2 with a high oxygen affinity were differentially expressed according to our proteomic study. Furthermore, enzymes for denitrification (NarGHIJ, NirS, NorCB, NosZ) or dissimilatory nitrate reduction (NarG, NIR) could not be found in the genome of our strains, except for *P. fragi* TMW2.2082, which encodes an assimilatory nitrate reductase, a small subunit of the nitrite reductase (NirD) as well as the enzyme NirE. However, we have not detected those proteins within the proteomic approach. Furthermore, our proteomic study revealed several enzymes for the usage of alternative electron donors to be strongly upregulated under anoxic conditions. The cytochrome bc1 complex and the protein cupredoxin were also upregulated under anoxic conditions, while cytochrome c and some other enzymes for usage of alternative electron donors were downregulated.

Figure 2B illustrates the genomically predicted arginine deiminase (ADI) pathway and enzymes differentially regulated according to our proteomic data. We identified ADI as well as the carbamate kinase to be upregulated under anoxic conditions while the agmatine deiminase was downregulated under anoxic conditions.

**FIGURE 3:**
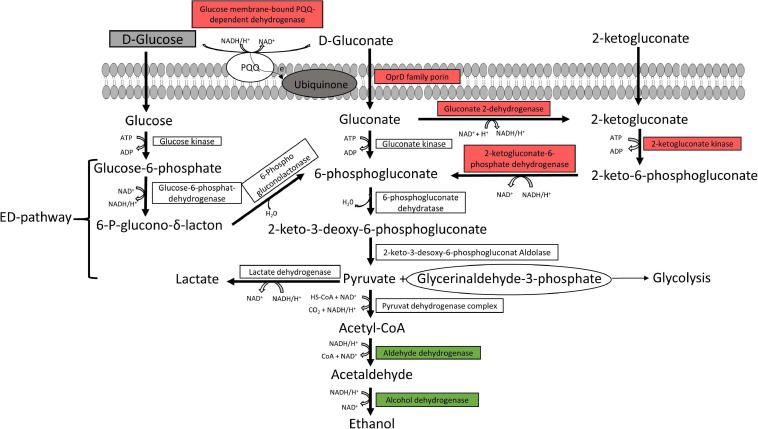
Predicted glucose and pyruvate metabolism. The glucose and pyruvate metabolism were predicted from the genome of the six strains *P. lundensis* TMW2.1732, *P. lundensis* TMW2.2076, *P. weihenstephanensis* TMW2.2077, *P. weihenstephanensis* TMW2.1728, *P. fragi* TMW2.2081, and *P. fragi* TMW2.2082, and differentially expressed enzymes based on our proteomic study were marked. ED, Entner-Doudoroff pathway. Enzymes marked in green were upregulated and enzymes marked in red downregulated under anoxic conditions for at least one strain.

The Entner-Doudoroff pathway and pyruvate metabolism are illustrated in Figure 3. Almost all enzymes responsible for gluconate and 2-ketogluconate uptake and degradation and the lactate dehydrogenase were downregulated under anoxic conditions. However, enzymes for glucose uptake and degradation to pyruvate were constitutively expressed under both conditions, while enzymes for the conversion of pyruvate to ethanol were upregulated under anoxic conditions.

Supplementary Figure 3 illustrates the proteins involved in tricarboxylic acid cycle and anaplerotic reactions, which were differentially expressed according to our proteomic data. The aconitase, isocitrate lyase, fumarase, glutaminase A, and glutamate dehydrogenase as well as enzymes of the BCKDH complex, poly-3-hydroxyalkanoate degradation and fatty acid beta oxidation were upregulated under anoxic conditions. Contrary, isocitrate dehydrogenase, succinyl-CoA synthase, asparaginase and aspartate ammonia-lyase were downregulated under anoxic conditions.

Complementary to the listed proteomic regulated enzymes and metabolic pathways in Figures 2, 3 and Supplementary Figure 3, several other differentially expressed enzymes were identified. Those comprise a downregulation of the ferrous metabolism, cellobiose and trehalose degradation, lipopolysaccharide and peptidoglycan synthesis and cell division under anoxic conditions for almost all strains. Contrary, degradation of maltose, sucrose, amylose, benzoate, RNA and murein as well as the glycine cleavage system, fatty acid biosynthesis, urease and oxidative stress proteins were upregulated under anoxic conditions for at least one strain.

### *In vitro* Test for Growth With NaNO_3_, Arginine, and Glucose

Anoxic growth with or without addition of NaNO_3_ was tested in yeast extract minimal media for all strains (Supplementary Table 3). *P. aeruginosa* DSM 1117 was the only strain exhibiting significant growth with sodium nitrate under anoxic conditions. Furthermore, all strains exhibited a negative API 20 NE test regarding reduction of nitrate to nitrite and nitrate to nitrogen (Supplementary Table 4).

All strains were able to grow aerobically and anaerobically on agar plates supplemented with L-arginine (Supplementary Figure 4). This was indicated by a color change from yellow to purple by L-arginine fermentation to ammonia for all strains. Plates incubated oxically were completely purple, while plates incubated anoxically only showed purple color next to the inoculation drops. Growth but no purple color could be observed for all strains cultivated aerobically on plates without arginine. All strains were unable to grow anaerobically on plates without arginine. Furthermore, the arginine deiminase test (ADH) of the API 20 NE was positive for all strains.

No growth and color change could be observed for all our strains on plates containing glucose monohydrate under anoxic conditions (Supplementary Figure 4). Oxically cultivated plates exhibited also no color change but enhanced growth on plates with glucose compared to the growth on plates lacking glucose.

Table 3 summarizes the results of the three physiological assays nitrate respiration, arginine fermentation and glucose metabolism obtained for all analyzed meat strains and *P. aeruginosa* DSM 1117. Further results of the API 20 NE test can be seen in the Supplementary Table 4 but are not discussed in more detail in this study, as they are not a part of the analyzed topic.

**TABLE 3:** Summary of *in vitro* experiments for identification of the main energy metabolism of *Pseudomonas* strains on meat.

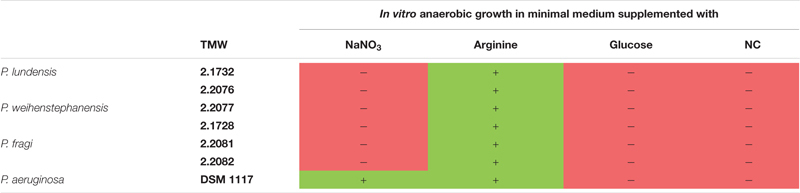

*Anaerobic growth of the strains P. lundensis TMW2.1732, P. lundensis TMW2.2076, P. weihenstephanensis TMW2.2077, P. weihenstephanensis TMW2.1728, P. fragi TMW2.2081, and P. fragi TMW2.2082 was tested in minimal medium supplemented either with sodium nitrate (NaNO_3_), arginine, or glucose monophosphate. As a negative control (NC), growth in minimal media without any ingredient was tested.*

## Discussion

Beside well-studied *P. fluorescens*, the species *P. lundensis*, *P. weihenstephanensis* and *P. fragi* are known as main aerobic meat spoilers (Erichsen and Molin, 1981; Ercolini et al., 2011; Casaburi et al., 2015; Höll et al., 2016; Hilgarth et al., 2019; Papadopoulou et al., 2020). Recently, strains of those species have been isolated *in situ* from minced beef stored under high-oxygen MAP atmosphere at a timepoint of oxygen limitation and depletion, and anaerobic growth of the three meat-spoiling species was demonstrated *in vitro* for the first time (Hilgarth et al., 2019). However, the underlying metabolic response mechanisms remained unknown as well as if selected strains can actually grow on meat in the absence of oxygen or only persist. Consequently, this follow-up study was performed with selected *Pseudomonas* strains from the study of Hilgarth et al. (2019) in order to study the anaerobic growth of these meat spoilers in detail and identify metabolism regulation to anaerobiosis. Furthermore, this study aimed to provide detailed insight in the hitherto limited research on the anaerobic lifestyle of *Pseudomonas* species in general, by conducting a comparative full-proteomic approach.

### Anaerobic Growth of *Pseudomonas* Strains on Chicken Breast Filet

We detected restricted growth (cell division rate 0.4–0.8/day) for the analyzed strains of the species *P. lundensis* (TMW2.1732/TMW2.2076), *P. weihenstephanensis* (TMW2.2077/TMW2.1728) and *P. fragi* (TMW2.2081/TMW2.2082) *in situ* on chicken breast filet packaged with 100% N_2_ atmosphere. These division rates are unlikely to result in accelerated spoilage if the cell counts are low i.e., average level of initial contamination. However, if levels of *Pseudomonas* spp. are high e.g., due to previous oxic storage or an unusual high initial contamination, spoilage cannot be delayed by the anoxic packaging as the organisms are still metabolically active. In conclusion, anoxic packaging results in decelerated growth of meat spoiling *Pseudomonas* spp. compared to oxic conditions, but does not lead to a complete growth repression.

### Uncovering the Anaerobic Metabolism and Lifestyle of *Pseudomonas* Species

We identified several metabolic pathways differentially regulated by the presence and absence of oxygen, which were frequently shared in their regulation between the strains but also between the species. Those metabolic pathways are discussed in detail in the following sections.

#### Aerobic and Anaerobic Respiration

The respiratory chain is the main energy-producing metabolism of aerobic bacteria, required for cell division and growth. Under oxic conditions, oxygen serves as a terminal electron acceptor for aerobic bacteria including *Pseudomonas* species. However, under anoxic conditions, some species of this genus e.g., *P. aeruginosa* and *P. stutzeri* are capable of restricted growth by denitrification or dissimilatory nitrate reduction using NO_3_ as a terminal electron acceptor (Fewson and Nicholas, 1961; Carlson and Ingraham, 1983; Zannoni, 1989; Yoon et al., 2002; Wu et al., 2005; Line et al., 2014). This study reveals the meat-spoiling *Pseudomonas* species *P. lundensis*, *P. weihenstephanensis*, and *P. fragi* to lack corresponding enzymes for dissimilatory nitrate reduction and denitrification in their genomes. This observation was further complemented by the fact that supplementation of NO_3_ to minimal medium *in vitro* does not enable anaerobic growth of the bacteria in this study, and nitrate reduction was negative for all analyzed strains in the API 20 NE test. Beside this, anaerobic growth of *Pseudomonas* strains on meat based on nitrate respiration is rather unlikely, as the amount of nitrate on white and red meat is quite low (max. 0.2 mmol^∗^kg^–1^ on red meat) (Lammarino and Di Taranto, 2012). Thus, we conclude that the growth of our strains on chicken meat is not based on energy gained from anaerobic respiration. However, the observed upregulation of several enzymes for different electron donors as well as copper related proteins, which are known to be frequently involved in electron transfer (Dennison, 2005; Farver and Pecht, 2011) under anoxic conditions, can be explained by the attempt of the cells to keep an anaerobic respiratory chain running regardless if substrates/co-factors for their functionality are present or not.

#### Arginine Fermentation

Arginine conversion via the ADI pathway has been reported to be limited to weak biomass increase of *P. aeruginosa* under anoxic conditions (Vander Wauven et al., 1984). This is due to the conversion of arginine-derived carbamoyl phosphate to ammonia, carbon dioxide and ATP by the enzymes arginine deiminase, ornithine carbamoyltransferase and carbamate kinase. Regarding these meat-spoiling *Pseudomonas* strains, we observed an upregulation of the ADI and carbamate kinase enzyme for several of our strains, indicating enhanced arginine fermentation under anoxic conditions. For both *P. lundensis* strains, respective genes were not differentially but equally strong expressed under both conditions enabling to benefit from arginine degradation under oxic as well as under anoxic conditions.

We further demonstrated that this metabolism provides sufficient energy conservation for anaerobic growth of the strains by performing *in vitro* plate assays with or without addition of 20 mM arginine. As the amount of arginine in meat is way higher compared to the minimal medium (440 mmol^∗^kg^–1^ protein on beef and 344 mmol/kg protein on chicken) (Wladyka and Dawson, 1968; Ahmad et al., 2018) we hypothesize that arginine fermentation is one main source of energy for our *Pseudomonas* strains, which enables restricted growth also *in situ* on anoxic packaged meat.

#### Glucose and Pyruvate Metabolism

There are three possible ways of glucose uptake for *Pseudomonas* species comprising direct uptake of glucose or indirect uptake of glucose-derived gluconate and 2-ketogluconate, all being ultimately shunted into the Entner-Doudoroff pathway (Chavarría et al., 2013). Enzymes of the indirect uptake system via the glucose membrane-bound PQQ-dependent dehydrogenase, which is known to shuttle electrons into the respiratory chain (Umezawa et al., 2015), and the NADH/H^+^ producing gluconate 2-dehydrogenase, were down-regulated under anoxic conditions. However, all enzymes for the standard ED-pathway were constitutively expressed without regulation for all strains in our study, conserving one ATP molecule per molecule glucose via production of glyceraldehyde-3-phosphate entering glycolysis pathway. Therefore, the energy yield of this metabolic pathway is equal to arginine fermentation. However, fermentation of arginine is not coupled to the NADH + /NAD + pool, and therefore, no redox equivalents have to be reoxidized to maintain redox balance. This reoxidation can be predictively performed by glucose fermentation to ethanol, indicated by an upregulation of corresponding enzymes (aldehyde and alcohol dehydrogenases) under anoxic conditions. The observed upregulation of enzymes utilizing maltose, sucrose and amylose further support the assumption of glucose utilization under anoxic conditions, especially as glucose is mainly bound in di-, tri- and polysaccharides in meat (Komiyama et al., 2008; Koutsidis et al., 2008). However, our growth experiment under anoxic conditions demonstrated that sole glucose metabolism/fermentation does not conserve enough energy and therefore does not enable true growth of our *Pseudomonas* strains on meat. Nevertheless, we predict long term survival of our strains by glucose metabolism via the Entner-Doudoroff pathway and NAD^+^ recycling by ethanolic formation.

Another metabolic pathway enabling long term survival of *P. aeruginosa* under anoxic conditions is thought to be acetate fermentation via the two enzymes phosphotransacetylase and acetate kinase (Eschbach et al., 2004; Schreiber et al., 2006). However, genomes of our six analyzed strains do not encode those enzymes and therefore employment of acetate fermentation for survival of these *Pseudomonas* strains on meat can be excluded.

#### Glyoxylate Shunt

Interestingly, we measured an upregulation of enzymes involved in the glyoxylate shunt as well as a concomitant downregulation of bypassed enzymes of the tricarboxylic acid cycle for our strains under anoxic conditions. The glyoxylate shunt is commonly activated in bacteria under carbon-source limitation. In this bacteria, acetate and fatty acids-derived acetyl-CoA is shuttled into the glyoxylate shunt to obtain C4 compounds which are channeled into gluconeogenesis to synthesize C5 or C6 compounds (Maloy et al., 1980; Son et al., 2007). Indeed, we also observed an upregulation of several proteins involved in anoxic fatty acid beta oxidation, leucine and isoleucine degradation as well as poly-3-hydroxyalkanoate degradation, all yielding acetyl-CoA under anoxic conditions. Gluconeogenetic enzymes replacing irreversible reactions of the glycolysis were not differentially regulated for our strains. However, reduced or disabled respiration has been reported to be associated with an increase in the glyoxylate shunt, preventing NADH/H^+^ production via the bypassed enzymes isocitrate dehydrogenase, α-ketoglutarate dehydrogenase and succinate dehydrogenase (Tielen et al., 2013; Ahn et al., 2016; Meylan et al., 2017).

#### Protein Folding

The absence of alternative terminal electron acceptors and impaired respiration imply redox stress within the cell due to an imbalance of redox balance. Thus, cytoplasmatic proteins are kept predominantly in a reduced status and are therefore unable to perform their functionality (Glasser et al., 2014; Lai et al., 2016). Our proteomic data indicated that the DsbA enzyme, which is needed for correct protein folding, was upregulated under anoxic conditions. According to Collet and Bardwell (2002), protein folding takes place by shuttling electrons from the native unfolded peptide chain via DsbA and DsbB proteins to a terminal electron acceptor. As reoxidation of Dsb proteins seems to be impaired in our strains, incorrect folded proteins are generated, which have to be repaired by isomerization of existing S-S bonds. In this context, we detected several corresponding enzymes to be upregulated under anoxic conditions, comprising thioredoxin, peroxiredoxin, cysteine hydrolases, and S-S-oxide reductases. Interestingly, thioredoxin and peroxiredoxin are under control of the transcription factor OxyR in *P. aeruginosa*, which also regulates the expression of catalase enzymes (Wei et al., 2012). This might also explain the upregulation of the catalase PHII for three of our *Pseudomonas* stains under anoxic conditions.

## Conclusion

To our knowledge this study is the first one demonstrating limited growth of the three meat-spoiling species *P. lundensis*, *P. weihenstephanensis* and *P. fragi in situ* on anoxically packaged chicken meat. Furthermore, we were able to explain the observed anaerobic growth of our strains by uncovering their basic metabolic lifestyle applying a full genomic and comparative proteomic approach. In detail, we predicted arginine fermentation via the ADI pathway to provide the necessary energy for the growth on anoxically packaged meat. We also conclude that glucose fermentation to ethanol via the Entner-Doudoroff pathway contributes to long term survival under those conditions, but alone does not enable true growth. Furthermore, the studied strains are unable to conduct nitrate respiration or fermentation of glucose to acetate due to absence of respective genes, which has previously been described for other *Pseudomonas* species under anoxic conditions. Under carbon-limited conditions and anoxia, we predict that these strains use the glyoxylate shunt for gluconeogenesis, bypassing enzymes of the TCA cycle producing NADH+. Furthermore, we highlighted the consequences of redox stress triggered by imbalance of the redox equivalents and impaired respiration on protein folding processes, counteracted by overexpression of refolding enzymes. Thus, we also complemented the hitherto limited research on the general anaerobic metabolism of different *Pseudomonas* species.

## Data Availability Statement

The mass spectrometry proteomics data have been deposited to the ProteomeXchange Consortium via the PRIDE (Perez-Riverol et al., 2019) partner repository with the dataset identifier PXD023961.

## Author Contributions

SK designed the study, performed the experiments and data analysis, and wrote the first draft of the manuscript. MA performed the mass spectrometric data acquisition and MaxQuant search. MH helped to interpret the data and draft the manuscript, and supervised the work of SK. RV initiated the project and supervised the work of SK. All authors read and approved the final manuscript.

## Conflict of Interest

The authors declare that the research was conducted in the absence of any commercial or financial relationships that could be construed as a potential conflict of interest.

## Supplementary Material

Supplementary Figure 1 Growth of our *Pseudomonas* strains *in vitro* in MS media. Growth of the six strains *P. lundensis* TMW2.1732, *P. lundensis* TMW2.2076, *P. weihenstephanensis* TMW2.2077, *P. weihenstephanensis* TMW2.1728, *P. fragi* TMW2.2081, and *P. fragi* TMW2.2082 in MS medium under oxic (21% O_2_, 0.03% CO_2_) and anoxic (100% N_2_) conditions was monitored for 48 h. Dotted lines: anaerobic growth, solid lines: aerobic growth. Yellow dots represent the timepoint of sample taking for proteomics.

Supplementary Figure 2 PH development *in vitro* in MS media. The pH-value of the six strains (a) *P. lundensis* TMW2.1732, (b) *P. lundensis* TMW2.2076, (c) *P. weihenstephanensis* TMW2.2077, (d) *P. weihenstephanensis* TMW2.1728, (e) *P. fragi* TMW2.2081, and (f) *P. fragi* TMW2.2082 in MS medium under oxic (21% O_2_, 0.03% CO_2_) and anoxic (100% N_2_) conditions was recorded for 48 h. Dotted lines: anaerobic growth, solid lines: aerobic growth. All values are based on three independent replicates. Error bars represent standard error values.

Supplementary Figure 3 Predicted tricarboxylic acid cycle. The tricarboxylic acid cycle and reactions of the anaplerotic metabolism were predicted from the genome of the six strains *P. lundensis* TMW2.1732, *P. lundensis* TMW2.2076, *P. weihenstephanensis* TMW2.2077, *P. weihenstephanensis* TMW2.1728, *P. fragi* TMW2.2081, and *P. fragi* TMW2.2082, and differentially expressed enzymes based on our proteomic study were marked. Enzymes marked in green were upregulated and enzymes marked in red downregulated under anoxic conditions for at least on strain.

Supplementary Figure 4 Anaerobic arginine and glucose fermentation of *Pseudomonas* strains *in vitro* in minimal media. Arginine and glucose fermentation was demonstrated by a plate assay were the six strains *P. lundensis* TMW2.1732, *P. lundensis* TMW2.2076, *P. weihenstephanensis* TMW2.2077, *P. weihenstephanensis* TMW2.1728, *P. fragi* TMW2.2081, *P. fragi* TMW2.2082, and the positive control *P. aeruginosa* DSM 1117 were grown on agar plates containing a minimal medium supplemented with either 20 mM arginine or glucose monohydrate or none of those ingredients. The pH dye phenol red indicates a pH-increase by a color change from yellow to purple red, which is given by catabolism of the amino acid arginine. Plates were either incubated oxically or anoxically.

Supplementary Table 1 Detailed list of all significantly differential regulated proteins. A list of all significantly differential (*p* < 0.01, log_2_ > 2) regulated proteins of the strains *P. lundensis* TMW2.1732, *P. lundensis* TMW2.2076, *P. weihenstephanensis* TMW2.2077, *P. weihenstephanensis* TMW2.1728, *P. fragi* TMW2.2081 and *P. fragi* TMW2.2082 comparing growth under air (21% O_2_, 0.0% CO_2_) and anoxic (100% N_2_) conditions in meat simulation media is provided. Colors indicate the effect of regulation with  log_2_(diff.) = 2,  log_2_ (diff.) = 3,  log_2_ (diff.) = 4,  log_2_ (diff.) = 5,  log_2_ (diff.) = 6,  log_2_ (diff.) ≥ 6 for downregulated proteins and  log_2_ (diff.) = 2,  log_2_ (diff.) = 3,  log_2_ (diff.) = 4,  log_2_ (diff.) = 5,  log_2_ (diff.) = 6,  log_2_ (diff.) ≥ 6 for upregulated proteins under anoxic conditions. All data are based on three independent replicates. ^∗^Functional categories are based on NCBI annotation, TIGR annotation, SEED annotation, KEGG annotation and own research.

Supplementary Table 2 List of the genomic setup of important pathways of the analyzed *Pseudomonas* strains. All genomically encoded enzymes corresponding to the discussed pathways respiratory chain, Entner-Doudoroff pathway, pyruvate metabolism, arginine metabolism and TCA cycle are listed for the strains *P. lundensis* TMW2.1732, *P. lundensis* TMW2.2076, *P. weihenstephanensis* TMW2.2077, *P. weihenstephanensis* TMW2.1728, *P. fragi* TMW2.2081, and *P. fragi* TMW2.2082. All data are based on genomic analysis (NCBI annotation and manual blast search). Numbers given are the corresponding locus tags of NCBI annotated genes, ^∗^ indicates putative genes.

Supplementary Table 3 Growth with NaNO_3_ *in vitro* in minimal media. Anaerobic growth (OD_600_) of the six strains *P. lundensis* TMW2.1732, *P. lundensis* TMW2.2076, *P. weihenstephanensis* TMW2.2077, *P. weihenstephanensis* TMW2.1728, *P. fragi* TMW2.2081, *P. fragi* TMW2.2082, and the positive control *P. aeruginosa* DSM 1117 was tested in minimal media containing glucose as electron donor supplemented with 20 mM NaNO_3_ or without NaNO_3_. Significant differences between growth with or without NaNO_3_ were defined by a *t*-test with *p* < 0.01.

Supplementary Table 4 API 20 NE test performed for our *Pseudomonas* strains. Results obtained from the API NE 20 test for the strains *P. lundensis* TMW2.1732, *P. lundensis* TMW2.2076, *P. weihenstephanensis* TMW2.2077, *P. weihenstephanensis* TMW2.1728, *P. fragi* TMW2.2081, and *P. fragi* TMW2.2082 after 48 h.
