# Supplementary figures and images for: Comparative Proteomics Reveals the Anaerobic Lifestyle of Meat-Spoiling Pseudomonas Species
Source: Front Microbiol. 2021 Apr 6;12:664061. doi: 10.3389/fmicb.2021.664061 (PMC8055858; doi:10.3389/fmicb.2021.664061)

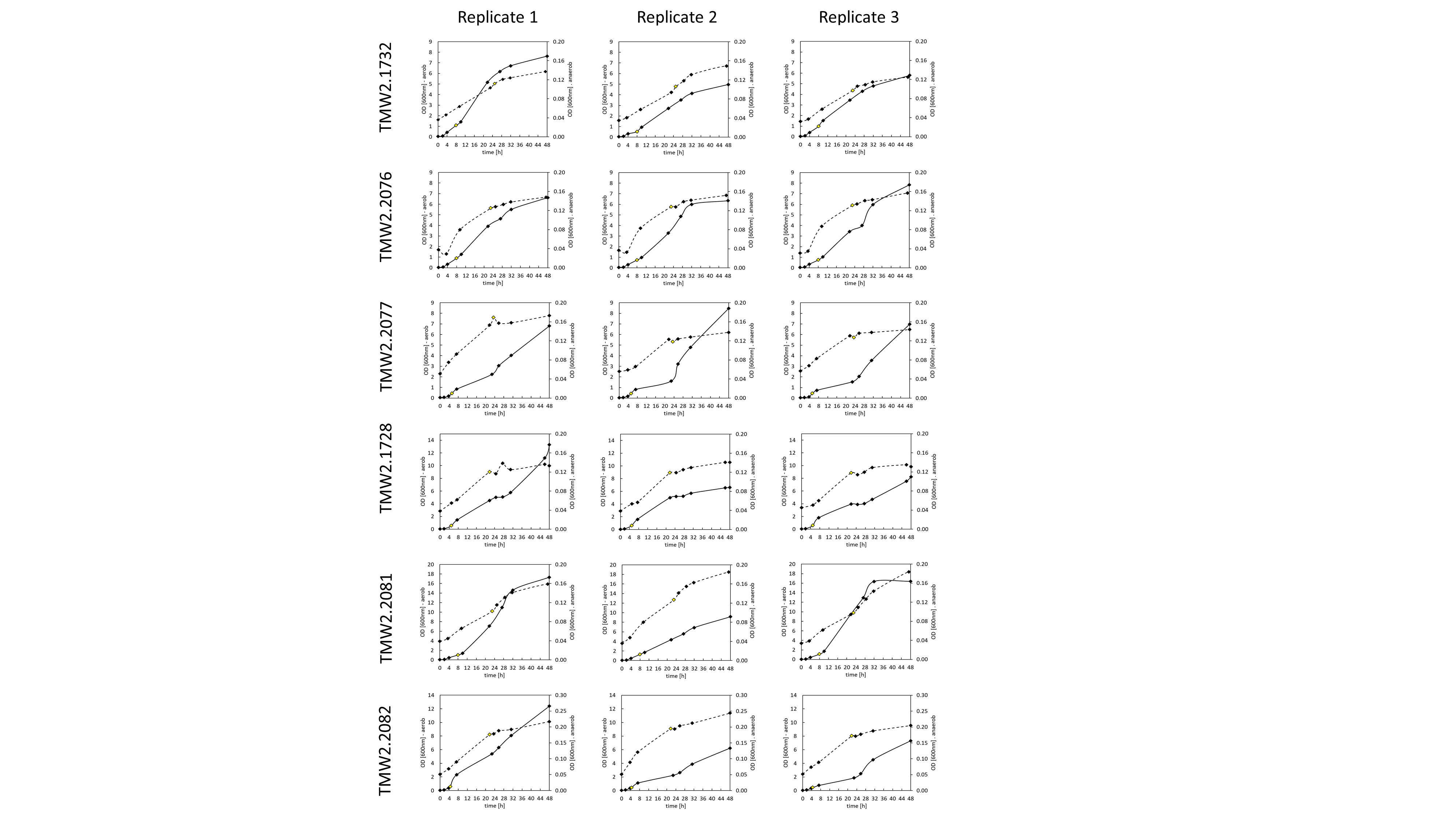

Supplement: Supplementary Figure 1 — Growth of our Pseudomonas strains in vitro in MS media. Growth of the six strains P. lundensis TMW2.1732, P. lundensis TMW2.2076, P. weihenstephanensis TMW2.2077, P. weihenstephanensis TMW2.1728, P. fragi TMW2.2081, and P. fragi TMW2.2082 in MS medium under oxic (21% O2, 0.03% CO2) and anoxic (100% N2) conditions was monitored for 48 h. Dotted lines: anaerobic growth, solid lines: aerobic growth. Yellow dots represent the timepoint of sample taking for proteomics. [file Image_1.TIF]

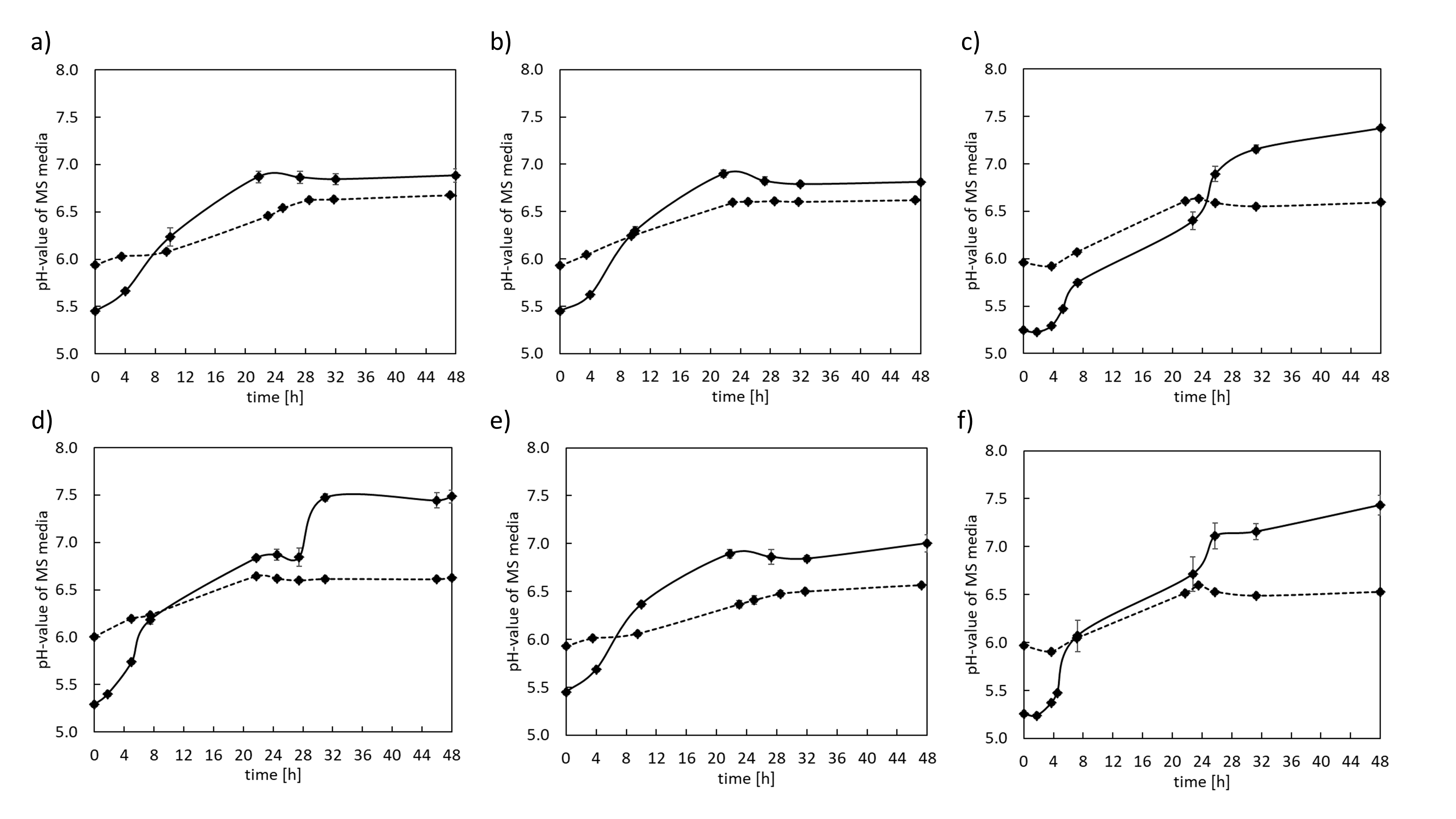

Supplement: Supplementary Figure 2 — PH development in vitro in MS media. The pH-value of the six strains (a) P. lundensis TMW2.1732, (b) P. lundensis TMW2.2076, (c) P. weihenstephanensis TMW2.2077, (d) P. weihenstephanensis TMW2.1728, (e) P. fragi TMW2.2081, and (f) P. fragi TMW2.2082 in MS medium under oxic (21% O2, 0.03% CO2) and anoxic (100% N2) conditions was recorded for 48 h. Dotted lines: anaerobic growth, solid lines: aerobic growth. All values are based on three independent replicates. Error bars represent standard error values. [file Image_2.TIF]

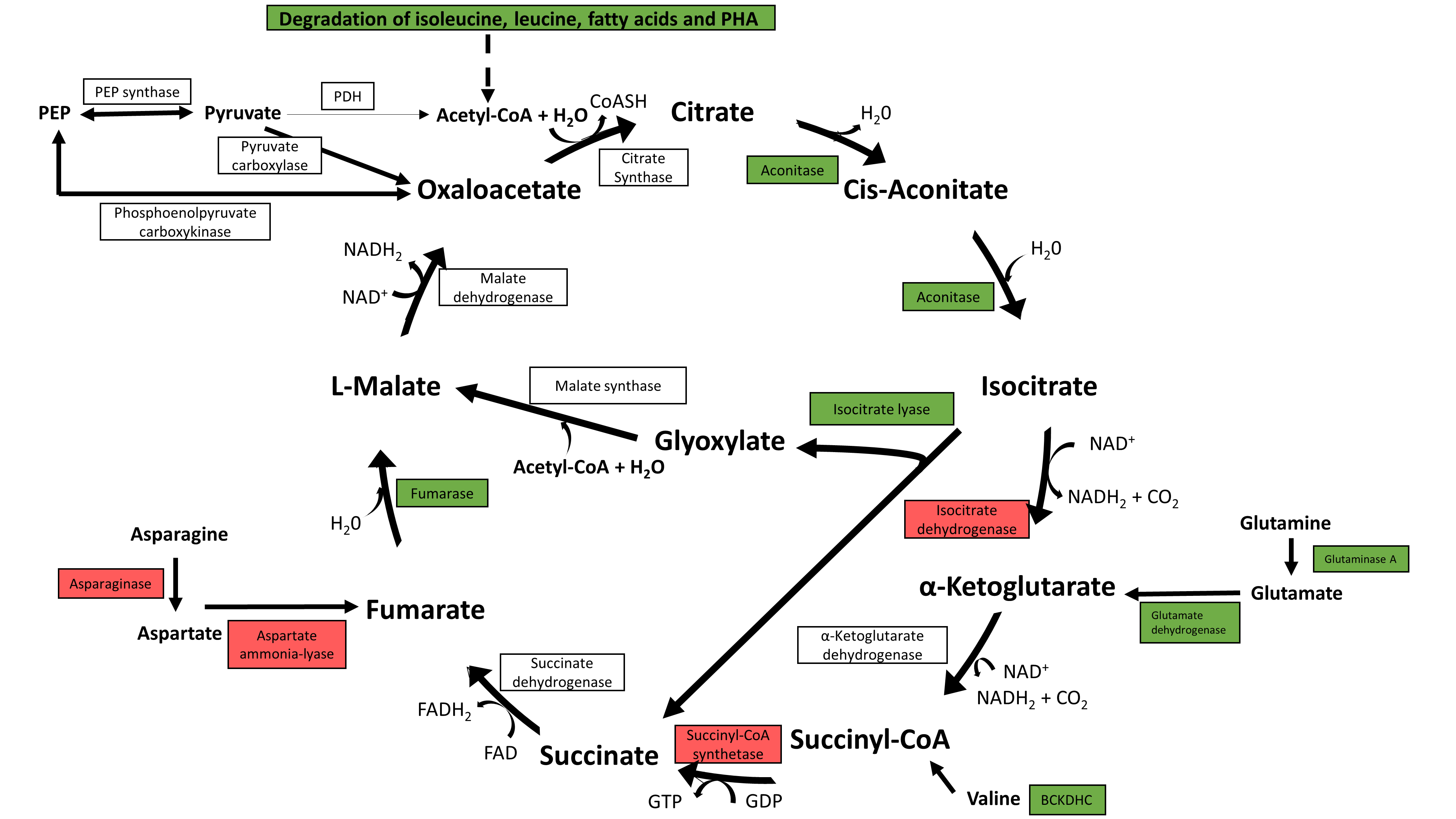

Supplement: Supplementary Figure 3 — Predicted tricarboxylic acid cycle. The tricarboxylic acid cycle and reactions of the anaplerotic metabolism were predicted from the genome of the six strains P. lundensis TMW2.1732, P. lundensis TMW2.2076, P. weihenstephanensis TMW2.2077, P. weihenstephanensis TMW2.1728, P. fragi TMW2.2081, and P. fragi TMW2.2082, and differentially expressed enzymes based on our proteomic study were marked. Enzymes marked in green were upregulated and enzymes marked in red downregulated under anoxic conditions for at least on strain. [file Image_3.tif]

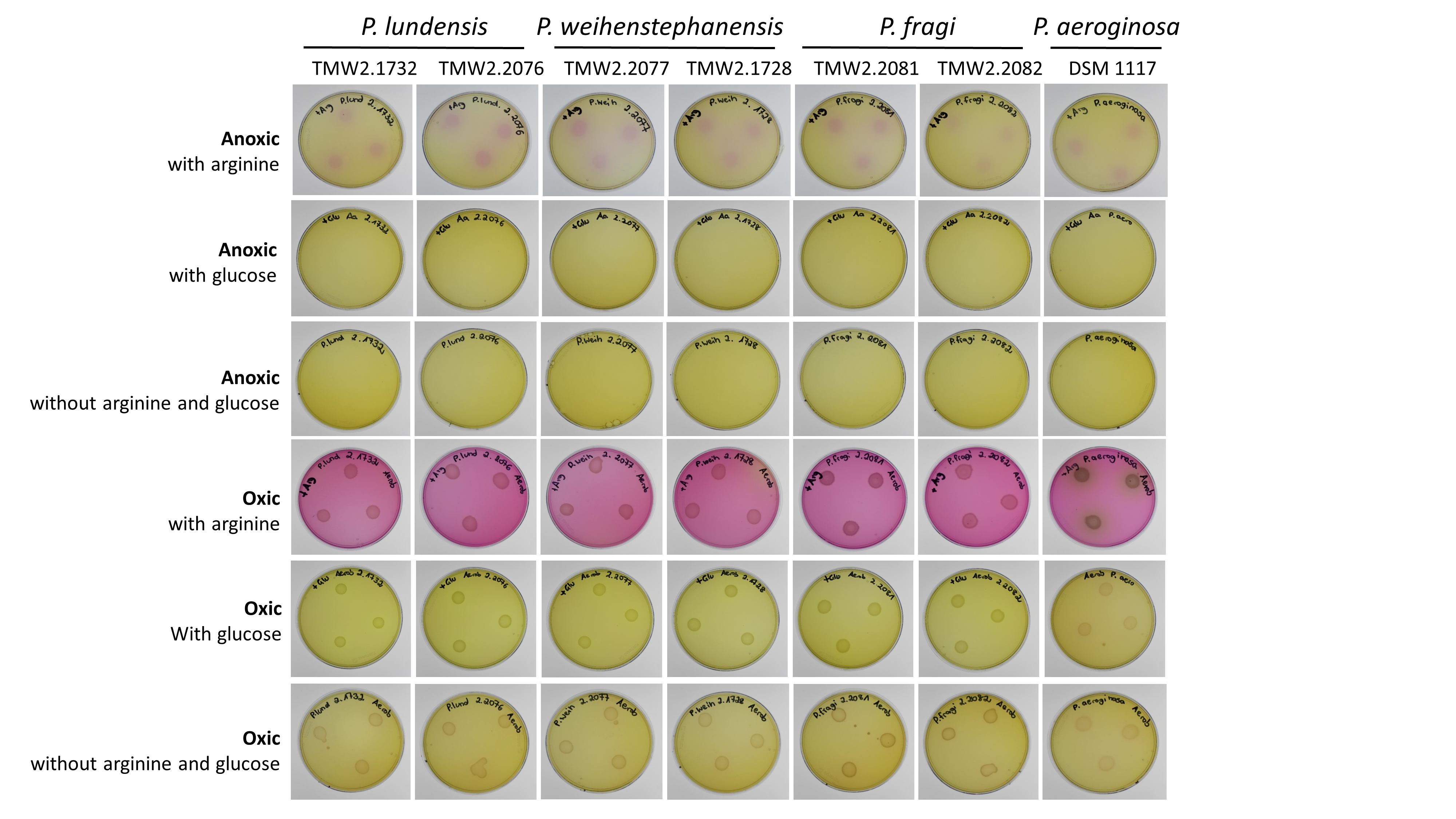

Supplement: Supplementary Figure 4 — Anaerobic arginine and glucose fermentation of Pseudomonas strains in vitro in minimal media. Arginine and glucose fermentation was demonstrated by a plate assay were the six strains P. lundensis TMW2.1732, P. lundensis TMW2.2076, P. weihenstephanensis TMW2.2077, P. weihenstephanensis TMW2.1728, P. fragi TMW2.2081, P. fragi TMW2.2082, and the positive control P. aeruginosa DSM 1117 were grown on agar plates containing a minimal medium supplemented with either 20 mM arginine or glucose monohydrate or none of those ingredients. The pH dye phenol red indicates a pH-increase by a color change from yellow to purple red, which is given by catabolism of the amino acid arginine. Plates were either incubated oxically or anoxically. [file Image_4.TIF]
