# Supplementary material for: Comparative Proteomics Reveals the Anaerobic Lifestyle of Meat-Spoiling Pseudomonas Species
Source: Front Microbiol. 2021 Apr 6;12:664061. doi: 10.3389/fmicb.2021.664061 (PMC8055858; doi:10.3389/fmicb.2021.664061)
Supplement: Supplementary Table 2 — List of the genomic setup of important pathways of the analyzed Pseudomonas strains. All genomically encoded enzymes corresponding to the discussed pathways respiratory chain, Entner-Doudoroff pathway, pyruvate metabolism, arginine metabolism and TCA cycle are listed for the strains P. lundensis TMW2.1732, P. lundensis TMW2.2076, P. weihenstephanensis TMW2.2077, P. weihenstephanensis TMW2.1728, P. fragi TMW2.2081, and P. fragi TMW2.2082. All data are based on genomic analysis (NCBI annotation and manual blast search). Numbers given are the corresponding locus tags of NCBI annotated genes, ∗ indicates putative genes. [file Table_2.docx]

|  |  | ***P. lundensis***  **TMW2.1732** | ***P. lundensis***  **TMW2.2076** | ***P. weihenstephanensis***  **TMW2.2077** | ***P. weihenstephanensis* TMW2.1728** | ***P. fragi***  **TMW2.2081** | ***P. fragi***  **TMW2.2082** |
| --- | --- | --- | --- | --- | --- | --- | --- |
| **Biosample** |  | **H4P02** | **GYM98** | **GYN02** | **GYN01** | **GYM96** | **GYM97** |
|  | | | | | | | |
| Respiratory chain | | | | | | | |
| Cytochrome o ubiquinol oxidase |  |  |  |  |  |  |  |
| Ubiquinol oxidase subunit II |  | 03220 | 01355 | 04970 | 05605 | 21085 | 14755 |
| Cytochrome o ubiquinol oxidase subunit I |  | 03225 | 01350 | 04975 | 05600 | 21080 | 14750 |
| Cytochrome o ubiquinol oxidase subunit III |  | 03230 | 01345 | 04980 | 05595 | 21075 | 14745 |
| Cytochrome o ubiquinol oxidase subunit IV |  | 03235 | 01340 | 04985 | 05590 | 21070 | 14740 |
| Cytochrome d ubiquinol oxidase | | | | | | | |
| Cytochrome ubiquinol oxidase subunit I |  | 02305 | 02280 | 04070 | 06530 | 13410 | 15655 |
| Cytochrome d ubiquinol oxidase subunit II |  | 02310 | 02275 | 04075 | 06525 | 13405 | 15650 |
| Ubiquinol-cytochrome c reductase iron-sulfur subunit |  | 2995 | 01550 | 04755 | 05820 | 21300 | 14970 |
| Cytochrome Cbb3 oxidases | | | | | | | |
| Cbb3-type cytochrome oxidase assembly protein |  | 08920 | 10565 | 09675 | 01775 | 04245 | 01950 |
| Cytochrome-c oxidase, cbb3-type subunit III |  | 08940 08960 | 10585 10605 | 09700 09720 | 01735 01755 | 04180 04195 04215 | 01900 01920 |
| Cbb3-type cytochrome c oxidase subunit 3 |  | 08945 08965 | 10590 10610 | 09705 09725 | 01730 01750 | 04190 04210* | 01895 01915* |
| Cytochrome-c oxidase, cbb3-type subunit II |  | 08950 08970 | 10595 10615 | 09710  09730 | 01725 01745 | 04185 04205 | 01890 01910 |
| Cytochrome-c oxidase, cbb3-type subunit I |  | 08955 08975 14095 | 10600 10620 13550 | 07675 09715 09735 | 12730 01720 01740 | 03470 04180 04200 | 01220 01885 01905 |
| Cytochrome c oxidase | | | | | | | |
| Cytochrome c oxidase subunit II |  | 07220 | 04610 | 02020 | 09500 | 08995 | 09785 |
| Cytochrome c oxidase subunit I |  | 07225 | 04605 | 02015 | 09495 | 08990 | 09790 |
| Cytochrome c oxidase subunit 3 |  | 07235 | 04595 | 02005 | 09485 | 08980 | 09800 |
| Cytochrome c oxidase assembly protein |  | 07230 | 04600 | 02010 | 09490 | 08985 | 09795 |
| Cytochrome c oxidase accessory protein |  | 08935 14215 | 10580 13665 | 07555 09690 | 12840 01760 | 03600 04230 | 01305 01935 |
| Cytochrome bc1 | | | | | | | |
| Cytochrome bc complex cytochrome b subunit |  | 3000 | 01545 | 04760 | 05815 | 21295 | 14965 |
| Cytochrome c1 |  | 03005 | 01540 | 04765 | 05810 | 21290 | 14960 |
| Cytochrome c | | | | | | | |
| Cytochrome c family protein |  | 03325 | 01255 | 05040 | 05530 | 20915 | 14585 |
| Cytochrome c |  | 07450 11565 21310 21605 | 03380 04390 21490 | 08625 11285 00705 | 08165 02835 03390 | 22855 08270 10175 | 18955 21215 23090 |
| Cytochrome b |  | 06425 08215 01135 | 03635 14835  19715 | 07735 07795 01015 | 08475 12610 12670 05305 | 01660 02420 10095 | 23105 00645 00645 |
| Pyrroloquinolin quinone biosynthesis | | | | | | | |
| Pyrroloquinoline quinone biosynthesis protein |  | 16705 | 18120 | 00270 | 07735 | 10345 | 8450 |
| Pyrroloquinoline quinone biosynthesis peptide chaperone |  | 16755 | 18125 | 00265 | 07730 | 10350 | 8445 |
| Pyrroloquinoline-quinone synthase |  | 16760 | 18130 | 00260 | 07725 | 10355 | 8440 |
| Pyrroloquinoline quinone biosynthesis protein |  | 16765 | 18135 | 00255 | 07720 | 10360 | 8435 |
| Pyrroloquinoline quinone precursor peptide |  | 16770 | 18140 | 00250 | 07715 | 10365 | 8430 |
| Pyrroloquinoline quinone biosynthesis protein |  | 16775 | 18145 | 00245 | 07710 | 10370 | 8425 |
| Nitrite reductase small subunit |  | - | - | - | - | - | 21440  21430 |
| Assimilatory nitrite reductase |  | - | - | - | - | - | 21445  21435 |
|  | | | | | | | |
| Entner-Doudoroff (ED) pathway | | | | | | | |
| Glucose-6-phosphat-dehydrogenase |  | 16090  15730 | 09340  07835 | 20440  14540 | 17350  16085 | 20210  15900 | 06060  04490 |
| 6-phosphogluconolactonase |  | 16085  15725 | 07840  09335 | 20435  14535 | 17345  16090 | 20205  15905 | 06055  04495 |
| Glucose/quinate/shikimate family membrane-bound PQQ-dependent dehydrogenase |  | 3635 | 00915 | 05330 17160 | 10715 05225 | 00430 07695 | 17570 20210 |
| Gluconate 2-dehydrogenase |  | 7460 | 04380 | 01730 | 09220 | 8685 | 10095 |
| Sugar kinase |  | 04810 | 06950  14025 | 07160  17365 | 13270  20710 | 00170  21870 | 19480  17830 |
| Decarboxylating 6-phosphogluconate dehydrogenase |  | 15725 | 9335 | 20435 | 16090 | 15905 | 04540 |
| Glucokinase |  | 16145 | 7780 | 14595 | 17405 | 20270 | 06120 |
| Phosphogluconate dehydratase |  | 16150 | 07775 | 14600 | 17410 | 20275 | 06125 |
| 2-dehydro-3-deoxy-phosphogluconate aldolase |  | 16080 | 07845 | 14530 | 17340 | 20200 | 06050 |
|  | | | | | | | |
| Embden-Meyerhof-Parnas (EMP) pathway | | | | | | | |
| Glucose-6-phosphate isomerase |  | 13475 | 17330 | 18735 | 19640 | 19125 | 21935 |
| 6-phosphofructokinase |  | - | - | - | - | - | - |
| Fructosebisphosphate-aldolase |  | 21265 | 03335 | 00660 | 08120 | 10215 | 08580 |
| Glycerinaldehyde-3-phosphate dehydrogenase |  | 21470 | 18580 | 11805 | 02970 | 05460 | 03190 |
| Phosphoglycerat kinase |  | 21275 | 03345 | 00670 | 08130 | 10205 | 08590 |
| Phosphoglycerate mutase |  | 00690 | 20185 | 07140 13705 | 15335 | 12090 | 13645 |
| Pyruvate kinase |  | 19250 | 08425 | 15910 | 16765 | 16840 | 05410 |
|  | | | | | | | |
| Pyruvate metabolism | | | | | | | |
| Pyruvate dehydrogenase complex | | | | | | | |
| Pyruvate dehydrogenase (acetyl-transferring), homodimeric (E1) |  | 01445 | 10915 | 13030 | 14690 | 11470 | 13030 |
| Dihydrolipoyllysine-residue acetyltransferase (E2) |  | 01440 | 10910 | 13035 | 14695 | 11475 | 13035 |
| Dihydrolipoyl-dehydrogenase E3 |  | 09325* | 09935* | 07115* | 02400* | 17185* | 19525* |
| Pyruvate formate lyase |  | - | - | - | - | - | - |
| Formate-nitrite transporter |  | 08185 | - | 01050 | 08510 | 10065 | 08715 |
| NADP^+^ aldehyde dehydrogenase |  | 06485 | 14895 | 02235  03690 | 06910  09710 | 00385  03735  05160  14105 | 01440  02905  16350  17615 |
| NAD(P) dependent alcohol dehydrogenase |  | 11500  04045 | 16520  06150 | 11220  17100 | 03455  10775 | 00315  06080 | 03590  17685 |
| Lactate dehydrogenase |  | 19845*  04085* | 14310*  17140*  17540* | 10410*  18940* | 04085*  19845* | 01240*  06735*  18925*  14490* | 21735*  04245*  07965*  16855* |
| Pyruvate oxidase (ubiquinone-dependent) |  | 22685 | 09445 | 08630 | 02830 | 05295 | 03040 |
| Acetate kinase |  | - | - | - | - | - | - |
|  | | | | | | | |
| Tricarboxylic acid (TCA) cycle | | | | | | | |
| Aconitate hydratase |  | 08995  19480 | 10640  08650 | 09755  16135 | 01700  16545 | 04160  16355 | 01865  04945 |
| Isocitrate lyase |  | 18765 | 10075 | 09185 | 02270 | 04710 | 02450 |
| Isocitrate dehydrogenase |  | 18720  18725 | 10115  10120 | 09225  09230 | 02225  02230 | 04670 | 02410 |
| Malate synthase |  | 16645 | 18015 | 12960 | 14620 | 10255 | 08540 |
| 2-oxoglutarate dehydrogenase |  | 18910  18915 | 09925  09930 | 09045  09050 | 02405  02410 | 04875  04880 | 02610  02615 |
| Succinyl-CoA synthetase |  | 18895  18900 | 09940  09945 | 09060  09065 | 02395  02390 | 04865  04860 | 02600  02595 |
| Succinate dehydrogenase |  | 18920  18925  18930  18935 | 09920  09915  09910  09905 | 09040  09035  09030  09025 | 02415  02420  02425  02430 | 04885  04890  04895  04900 | 02620  02625  02630  02635 |
| Fumarase (Fumarate hydratase) |  | 19235  20595  08570 | 10215  08410  15525 | 09325  15895  19450 | 16775  18970  02130 | 14870  16850  04555 | 05420  07585  02295 |
| Malate dehydrogenase |  | 01065 | 19785 | 13345 | 14980 | 11745 | 13300 |
| Citrate synthase |  | 18940 | 09900 | 09020 | 02435 | 04905 | 02640 |
|  | | | | | | | |
| Anaerobe fatty acid beta oxidation | | | | | | | |
| Long-chain fatty acid CoA ligase |  | 16045  16050 | 07880  07875 | 14495  14500 | 17305  17310 | 20170  20165 | 06015  06020 |
| 3-hydroxyacyl-CoA dehydrogenase |  | 13695* | 20810* | 18450* | 00555* | 22955* | 19055* |
| acetyl-CoA acyltransferase |  | 13690 | 20805 | 18455 | 00550 | 22950 | 19050 |
|  | | | | | | | |
| Poly-3-hydroxyalkanoate (PHA) degradation | | | | | | | |
| Poly(3-hydroxyalkanoate) granule-associated protein |  | 01015  01005 | 19835  19845 | 13400  13390 | 15025  15035 | 11795 | 13350 |
| Phasin family protein |  | 01020 | 19830 | - | - | 11790 | 13345 |
| 3-hydroxybutyrate dehydrogenase |  | 13665 | 20780 | 18480 | 00525 | 22925 | 19025 |
| CoA transferase |  | 12250  12255 | 00360  00365 | 05855  05860 | 04685  04690 | 07160  07165  19330  19335 | 20990  20985  18415  18420 |
| Acetyl-CoA C-acyltransferase |  | 13690 | 20805 | 18455 | 00550 | 22950 | 19050 |
|  | | | | | | | |
| Anaplerotic reactions | | | | | | | |
| Asparaginase |  | 20245  09105 | 05640  10745 | 02940  09865 | 01590  10525 | 21325  21900  22670 | 14995  19450  22935 |
| Aspartate ammonia-lyase |  | 20230 | 05655 | 02950 | 10540 | 22660 | 22945 |
| Branched-chain alpha-keto acid dehydrogenase complex | | | | | | | |
| Alpha-ketoacid dehydrogenase E1 |  | 18915 | 09925 | 09045 | 02410 | 04880 | 02615 |
| 2-oxo acid dehydrogenase subunit E2 |  | 18910 | 09930 | 09050 | 02405 | 04875 | 02610 |
| Dihydrolipoyl dehydrogenase E3 |  | 18905 | 09935 | 09055 | 02400 | 04870 | 02605 |
| Glutamate dehydrogenase |  | 02390  06570 | 14980  02195 | 04155  07865 | 12540  06445 | 02360  13325 | 00560  15565 |
| Glutaminase |  | 02600 | 21630  01985 | 04370 | 06235 | 13115 | 15355 |
|  | | | | | | | |
| Gluconeogenesis | | | | | | | |
| Phosphoenolpyruvate carboxykinase |  | 00855 | 20350 | 13550 | 15185 | 11945 | 13500 |
| Phosphoenolpyruvate synthase |  | 11190 | 16830 | 10720 | 03780 | 06390 | 03900 |
| Fructose-1,6-bisphosphatase |  | 00765 | 20260 | 13635 | 15265 | 12020 | 13575 |
|  | | | | | | | |
| Arginine metabolism | | | | | | | |
| Arginine ornithine antiporter |  | 16195  16190 | 07730  07735 | 14640  14645 | 17450  17455 | 20315  20320 | 06170  06165 |
| Arginine deiminase |  | 16200 | 07725 | 14650 | 17460 | 20325 | 06175 |
| Ornithine carbamoyltransferase |  | 16205 | 07720 | 14655 | 17465 | 20330 | 06180 |
| Carbamate kinase |  | 16210 | 07715 | 14660 | 17470 | 20335 | 06185 |
| Arginine decarboxylase |  | 14795 | 11780 | 12170 | 13855 | 10670 | 12230 |
| Agmatine deiminase |  | 00475 | 19000 | 13935 | 15555 | 12395 | 13905 |
