# Supplementary material for: Comparative Proteomics Reveals the Anaerobic Lifestyle of Meat-Spoiling Pseudomonas Species
Source: Front Microbiol. 2021 Apr 6;12:664061. doi: 10.3389/fmicb.2021.664061 (PMC8055858; doi:10.3389/fmicb.2021.664061)
Supplement: Supplementary Table 3 — Growth with NaNO3 in vitro in minimal media. Anaerobic growth (OD600) of the six strains P. lundensis TMW2.1732, P. lundensis TMW2.2076, P. weihenstephanensis TMW2.2077, P. weihenstephanensis TMW2.1728, P. fragi TMW2.2081, P. fragi TMW2.2082, and the positive control P. aeruginosa DSM 1117 was tested in minimal media containing glucose as electron donor supplemented with 20 mM NaNO3 or without NaNO3. Significant differences between growth with or without NaNO3 were defined by a t-test with p < 0.01. [file Table_3.docx]

|  | | **with NaNO_3_** | | **without NaNO_3_** | | **p-value** |
| --- | --- | --- | --- | --- | --- | --- |
| TMW | | mean | SE | mean | SE |  |
| *P. lundensis* | 2.1732 | 0.01 | 0.00 | 0.03 | 0.01 | 0.346 |
| *P. lundensis* | 2.2076 | 0.00 | 0.00 | 0.00 | 0.00 | 0.856 |
| *P. weihenstephanensis* | 2.2077 | 0.01 | 0.00 | 0.01 | 0.00 | 0.848 |
| *P. weihenstephanensis* | 2.1728 | 0.00 | 0.00 | 0.00 | 0.00 | 0.783 |
| *P. fragi* | 2.2081 | 0.01 | 0.00 | 0.00 | 0.00 | 0.026 |
| *P. fragi* | 2.2082 | 0.00 | 0.00 | 0.01 | 0.01 | 0.345 |
| *P. aeruginosa* | DSM 1117 | 0.12 | 0.02 | 0.02 | 0.00 | 0.008 |
