# Supplementary material for: Comparative Proteomics Reveals the Anaerobic Lifestyle of Meat-Spoiling Pseudomonas Species
Source: Front Microbiol. 2021 Apr 6;12:664061. doi: 10.3389/fmicb.2021.664061 (PMC8055858; doi:10.3389/fmicb.2021.664061)
Supplement: Supplementary Table 4 — API 20 NE test performed for our Pseudomonas strains. Results obtained from the API NE 20 test for the strains P. lundensis TMW2.1732, P. lundensis TMW2.2076, P. weihenstephanensis TMW2.2077, P. weihenstephanensis TMW2.1728, P. fragi TMW2.2081, and P. fragi TMW2.2082 after 48 h. [file Table_4.docx]

|  | **TMW** | **NO_3_** | **TRP** | **GLU** | **ADH** | **URE** | **ESC** | **GEL** | **PNPG** | **GLU** | **ARA** | **MNE** | **MAN** | **NAG** | **MAL** | **GNT** | **CAP** | **ADI** | **MLT** | **CIT** | **PAC** | **OX** |
| --- | --- | --- | --- | --- | --- | --- | --- | --- | --- | --- | --- | --- | --- | --- | --- | --- | --- | --- | --- | --- | --- | --- |
|  | | | | | | | | | | **Assimilatory tests** | | | | | | | | | | | | |
| *P. lundensis* | 2.1732 | - | - | - | + | + | - | - | - | + | + | - | - | - | - | + | - | - | + | + | - | + |
|  | 2.2076 | - | - | - | + | + | - | + | - | + | + | - | - | - | - | + | - | - | + | + | - | + |
| *P. weihenstephanensis* | 2.2077 | - | - | - | + | - | - | - | - | + | - | - | - | - | - | + | - | - | + | + | - | + |
|  | 2.1728 | - | - | - | + | - | - | - | - | + | - | - | - | - | - | + | - | - | + | + | - | + |
| P. fragi | 2.2081 | - | - | - | + | + | - | - | - | + | + | - | - | - | - | + | - | - | + | + | - | + |
|  | 2.2082 | - | - | - | + | + | - | - | - | + | + | - | - | - | - | + | - | - | + | + | - | + |
